# CARGEL Bioscaffold improves cartilage repair tissue after bone marrow stimulation in a minipig model

**DOI:** 10.1186/s40634-020-00245-7

**Published:** 2020-05-08

**Authors:** K. Hede, B. B. Christensen, M. L. Olesen, J. S. Thomsen, C. B. Foldager, M. C. Lind

**Affiliations:** 1 Orthopedic Research Laboratory, Aarhus University Hospital, Palle Juul-Jensens Boulevard 99, Section J, Level 1, 8200 Aarhus N, Denmark; 2 Department of Biomedicine, Aarhus University, Wilhelm Meyers Allé 3, 8000 Aarhus C, Denmark; 3 Sports Trauma Clinic, Aarhus University Hospital, Palle Juul-Jensens Boulevard 99, Section J, Level 1, 8200 Aarhus N, Denmark

**Keywords:** Articular cartilage, Cartilage repair, Knee, Bone marrow stimulation, Microfracture, Drilling, Minipig

## Abstract

**Purpose:**

To gain knowledge of the repair tissue in critically sized cartilage defects using bone marrow stimulation combined with CARGEL Bioscaffold (CB) compared with bone marrow stimulation (BMS) alone in a validated animal model.

**Methods:**

Six adult Göttingen minipigs received two chondral defects in each knee. The knees were randomized to either BMS combined with CB or BMS alone. The animals were euthanized after 6 months. Follow-up consisted of histomorphometry, immunohistochemistry, semiquantitative scoring of the repair tissue (ICRS II), and μCT of the trabecular bone beneath the defect.

**Results:**

There was significantly more fibrocartilage (80% vs 64%, *p* = 0.04) and a trend towards less fibrous tissue (15% vs 30%, *p* = 0.05) in the defects treated with CB. Hyaline cartilage was only seen in one defect treated with CB and none treated with BMS alone.

For histological semiquantitative score (ICRS II), defects treated with CB scored lower on subchondral bone (69 vs. 44, *p* = 0.04). No significant differences were seen on the other parameters of the ICRS II. Immunohistochemistry revealed a trend towards more positive staining for collagen type II in the CB group (*p* = 0.08). μCT demonstrated thicker trabeculae (*p* = 0.029) and a higher bone material density (*p* = 0.028) in defects treated with CB.

**Conclusion:**

Treatment of cartilage injuries with CARGEL Bioscaffold seems to lead to an improved repair tissue and a more pronounced subchondral bone response compared with bone marrow stimulation alone. However, the CARGEL Bioscaffold treatment did not lead to formation of hyaline cartilage.

## Introduction

Cartilage lesions are common and do not heal spontaneously due to the avascular and aneural nature of the tissue. Cartilage lesions can lead to pain and early osteoarthritis [1]. Bone marrow stimulation techniques (BMS) such as microfracture (Mfx) is the preferred treatment option for small, symptomatic cartilage lesions in the knee [2]. The rationale behind BMS is to allow bone marrow mesenchymal stem cells to migrate to the lesion and to induce and facilitate a repair response. This treatment is surgically time-efficient, inexpensive, and have good short-term outcome. The repair response, however, consists primarily of fibrocartilage and fibrous tissue, which does not possess the same biomechanical properties as hyaline cartilage and is therefore more susceptible to wear causing deterioration of the early results [3]. While BMS can be a good treatment option for very small lesions augmentation is needed for larger lesions. The most common strategy for augmentation of BMS is to combine the procedure with cell-free scaffolds that may facilitate cartilage repair biomechanically and biologically. Numerous products have been introduced to the market, but early literature on their use is of limited quality [4].

Cartilage repair by Mfx is initiated by bone marrow-derived cells found in the blood clot, which fills the defect following penetration of the subchondral bone. Differences in blot clot stability may explain differences in repair tissue outcomes. CARGEL Bioscaffold (CB) (formerly BST CarGel; Smith & Nephew) is a chitosan-based biomaterial used as an adjuvant to bone marrow stimulation. It has mostly been used with a mini-arthrotomy, but it has also been proposed for arthroscopic techniques [5]. The purpose of the chitosan scaffold is stabilization of the bone marrow clot in the cartilage lesion after bone marrow stimulation to allow formation of improved repair tissue [6–8]. CB combined with Mfx has proven safe and has shown superior repair tissue quantity and quality compared to Mfx after 5 years [9–11]. CB has also been used for cartilage lesions in the hip where it has also shown safety of use and superior patient outcomes compared with Mfx alone [12]. Furthermore, use of CB has been suggested to be a cost-saving alternative to Mfx due to greater improvements in the induction of cartilage repair tissue with hyaline characteristics [13]. However, clinical studies are limited in characterizing the biological and morphological characteristics of the repair tissue, which is one of the main predictors of long-term outcome. Animal studies of CB have mainly been conducted in smaller animal models where there is a tendency to spontaneous healing of cartilage defects [7, 8, 14–17].

The aim of this study was to investigate and compare the morphological and histological effects of a combination of BMS and CB with BMS alone in chondral defects in the knees of Göttingen minipigs. The hypothesis was that treatment with CB would improve repair tissue quality compared with BMS.

## Materials and methods

### Experimental design

Six skeletally mature male Göttingen minipigs (weighing 38.4 kg, range 36.4–43.6 kg; aged 19.4 months, range 18.9–21.1 months) were included in the study. Two cylindrical chondral defects were created in the trochlea of each knee with a diameter of 6 mm, which has been shown to be a critical size defect in Göttingen Minipigs [18]: One defect in the medial trochlear facet and one defect in the lateral trochlear facet. The defects of each knee were randomized to treatment with either marrow stimulation or marrow stimulation in combination with CB (Smith & Nephew, Hørsholm, DK). The animals were euthanized after 6 months. Follow-up consisted of μCT, histomorphometry, semiquantitative scoring of histology (International Cartilage Repair Society [ICRS II]), and immunohistochemistry.

The study was conducted according to the Danish Law on Animal Experimentation and approved by the Danish Ministry of Justice Ethical Committee (J.nr. 2017-15-0201-01343).

### Surgery

The Göttingen mini-pig animal model has previously been described in detail [19–21]. Animals were premedicated with Zoletil Mix 1 mL/10 kg (tiletamin 2.5 mg/mL, zolazepam 2.5 mg/mL, torbugesic 0.5 mg/mL, ketaminol 2.5 mg/mL, and rompun 2.5 mg/mL; Virbac, DK). General anesthesia and local analgesia were achieved with Etomidate (Hypnomidate, 0.25 mL/kg; Janssen Pharmaceuticals), sevoflurane (3%; AbbVie), fentanyl (0.175 mL/kg/h, Hameln Pharmaceuticals), and Lidocaine (Xylocaine 10 mL, 20 mg/mL; Astra Zeneca). Preoperative prophylactic antibiotics were used (penicillin procaine, 0.03 mL/kg; Ceva Sante Animale, France).

Access to the knee joint was gained through the patellar ligament. The trochlea was exposed, and two chondral defects with a diameter of 6 mm were created using a skin biopsy punch and a curette. One defect was made in the distal, medial trochlea, while the other was made in the lateral trochlea, 0.5 to 1 cm proximal to the first defect. This was done in all knees, and both defects in each knee were treated with the same method. The defects were thoroughly debrided by use of a curette, and the calcified cartilage layer was carefully removed in order not to damage the subchondral bone.

**Fig. 1:**
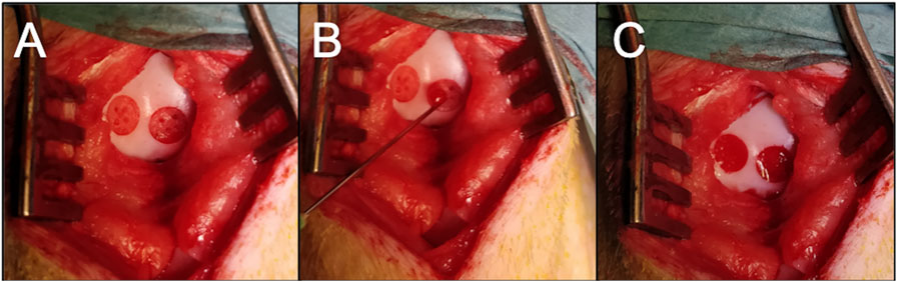
**a** shows the defects after debridement, drilling and drying. **b** shows application of the CARGEL. **c** is immediately after the application

In the BMS group, ﻿four holes (depth 5 mm, diameter 1 mm) were drilled into the subchondral bone, and bleeding from the bone marrow was observed. In the group with marrow stimulation combined with CB, bone marrow stimulation was performed as described above. The defect was then dried using a small swab (Fig. 1a). Meanwhile, the CB was prepared according to manufacturer’s instructions with 4.5 mL of autologous venous blood, drawn from the ear vein. The leg was positioned to ensure a horizontal position of the defect. The CB was then injected, and the defect filled entirely with care taken not to overfill the defect (Fig. 1b+C). The clot was allowed 15 min to stabilize and the knee was put through 40 full range of motion movements followed by visual inspection to ensure the clot was still in place.

After treatment﻿, the patella ligament, subcutaneous tissue and skin were sutured, and subcutaneous lidocaine was injected for pain management. The animals ﻿were treated postoperatively with Finadyne 5% (Flunixin meglumin, 1.1 mg/kg, oral paste, Intervet, Denmark) for 5 days and were allowed immediate weight-bearing and full-range of motion postoperatively. ﻿Trained animal keepers, supervised by a veterinarian, closely observed each animal thrice daily. After 6 months the animals were euthanized using Pentobarbital (0.4 mL/kg) and osteochondral blocks of 1 cm × 1 cm × 1 cm around the defect were cut for further analyses.

### Preparation of specimens

The samples were prepared as previously described in detail [22]. In brief, samples were dehydrated in ethanol of increasing concentration (70%–96%) and cleared in isopropanol and xylene. The samples were then embedded in methyl methacrylate (MMA).

### μCT

The MMA embedded osteochondral blocks were scanned in a desktop μCT scanner (Scanco μCT 35; Scanco Medical, Brüttiselen, Switzerland) with an isotropic voxel size of 10 μm, X-ray voltage of 55 kV, current of 145 μA, and an integration time of 800 ms in high resolution mode (1000 projections/180°). The trabecular bone was analyzed by drawing a 2-mm-high cylindrical VOI (volume of interest) with a diameter of 6 mm in the trabecular bone beneath the defect using a custom-made computer program running under Linux [23]. The VOI was imported into the software provided with the scanner (IPL version 6.5). The 3D data sets were low-pass filtered using a Gaussian filter (σ = 1.3, support = 2) in order to remove noise before segmentation with a fixed threshold filter (threshold = 510.3 mg HA/cm^3^).

Analyses included bone volume fraction (BV/TV), trabecular thickness (Tb.Th), trabecular number (Tb.N), trabecular separation (Tb.Sp), connectivity density (CD), structure model index (SMI), and bone material density (ρ).

### Histologic evaluation

The MMA embedded osteochondral blocks were cut into 7-μm-thick sections by use of a hard tissue microtome (Reichert Jung Polycot). All samples were stained with hematoxylin and eosin, safranin O, toluidine blue and immunohistochemically stained for collagen type I or II. All evaluations were done by a single, blinded assessor with experience in experimental cartilage repair.

### Histomorphometry

The morphological characteristics of the repair tissue were quantitatively evaluated by means of histomorphometry as described by Foldager et al. [24]. Each defect was halved, and sections were cut for every 350 μm, yielding 7 sections per defect. A 5 × 5 point counting grid was superimposed onto each section at × 10 magnification (newCAST software; Visiopharm), and 50% of the defect was counted according to tissue type (hyaline cartilage, fibrocartilage, fibrous tissue, bone, or vascular tissue) as previously described in detail using hematoxylin and eosin staining with polarized light to accentuate collagen fibers [22]. Hyaline cartilage was defined as rounded cells in lacunae within a hyaline matrix, fibrocartilage as rounded cells in lacunae within a fibrous matrix and fibrous tissue as elongated cells in a fibrous matrix.

Metachromasia on safranin-O staining was furthermore analyzed on a single, central section per defect and the percentage of repair tissue with metachromasia was determined [8, 17].

### Semiquantitative scoring

Blinded evaluation of a central section of each defect by use of the ICRS II histological score was performed. In the ICRS II score, the sections are evaluated with a visual analog scale from 0 (severely abnormal) to 100 (normal) compared to normal hyaline cartilage. There are 14 categories: tissue morphological characteristics, matrix staining, cell morphological characteristics, chondrocyte clustering, surface architecture, basal integration, formation of a tidemark, subchondral bone abnormalities, inflammation, abnormal calcification, vascularization, surface assessment, deep zone assessment, and overall assessment [20]. Safranin O and toluidine blue staining was used for the ICRS II evaluation.

### Immunohistochemistry

Immunohistochemical staining was performed on central sections with polyclonal rabbit antibodies for collagen type I (Abcam, Ab 34,710, Cambridge, UK) and collagen type II (Neomarkers, MS 306-P0, Fremont, CA) was performed as previously described using a Dako Autosatiner (Dako Universal Staining System, Carpinteria, CA) [25]. Negative staining controls were labeled with rabbit serum (Dako, X0902) or mouse IgG isotype control (Thermo Fisher Scientific, Camarillo, CA), respectively. For labeling streptavidin-horse radish peroxidase and aminoethyl carbazole was used according to the manufacturer’s instructions (Dako). The sections were counterstained with Mayer’s hematoxylin. A visual analog scale (0–100) was used to evaluate the percentage of positively stained repair tissue for collagen type I and II for each sample [20]. The amount of positively stained tissue was ranged in quartiles from 0 to 25%, 25–50%, 50–75% or 75–100%.

### Statistical analysis

Sample size was determined by power analysis based on overall ICRS II score as primary endpoint. Based on recent studies we expected that BMS without enhancement would score 10 points and enhanced BMS would score 30 points. SD for the ICRS II score was expected to be 15 points. Power was set to 80%, α = 0.05 and β = 0.2. With these assumption nine treatment units per study group was needed. We decided to include 12 units per group to account for possible animal dropout.

For measures of cartilage repair (histomorphometry and histology (ICRS score)), a mixed-effect model was fitted to the data, with pig and knee (left or right) treated as random effects, and treatment (BMS or CB) and defect site (proximal/lateral or distal/medial) as fixed effects. A *p-*value of less than 0.05 was considered significant. Each category from the ICRS II score was analyzed separately. μCT data was compared using unpaired t-tests. The number of samples in each quartile of positive collagen staining was analyzed using Fisher’s exact test. Two-tailed *p*-values less than 0.05 were considered significant. Statistical analysis was performed using STATA version 15.0 (StataCorp, College Station, TX, USA) and Prism 7 (GraphPad Software, Inc.).

## Results

All animals went through 6 months follow-up and there were no per- or postoperative complications.

Repair tissue was irregular and opaque and was easily distinguishable from the native cartilage in both treatment groups (supplementary Fig. 1 + 2). One defect/specimen from each group was lost due to theft while being transported for μCT.

### μCT

**Fig. 2:**
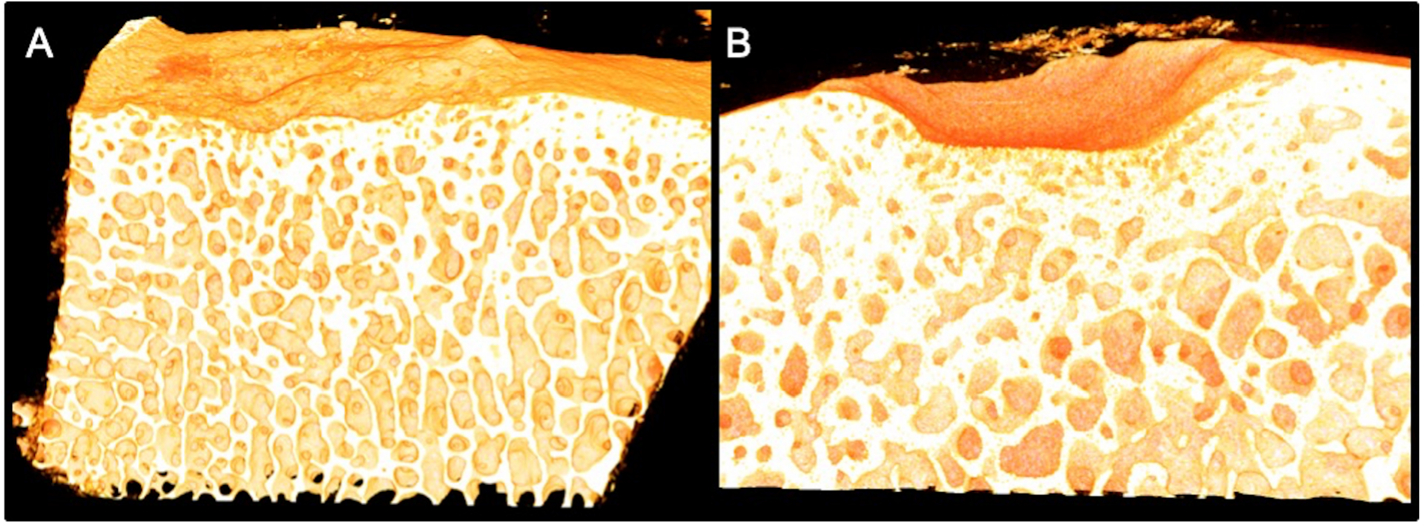
**a** shows a μCT image of a defect treated with BMS alone showing almost completely restored subchondral bone plate. **b** shows a defect treated with CB with pronounced subchondral remodeling

Defects treated with CB had significantly thicker trabeculae (170 μm vs. 150 μm, *p* = 0.029) and the bone had significantly higher bone material density (861 mg HA/cm^3^ vs. 845 mg HA/cm^3^, *p* = 0.028) indicating that the bone was more mineralized (Table 1 and Fig. 2).

**Table 1:** μCT data ± standard deviations. * and bold = parameters with significant differences. BMS = bone marrow stimulation

Parameter	BMS	BMS + CARGEL
Bone volume fraction (BV/TV)	0.471 ± 0.01	0.499 ± 0.07
Connectivity density (CD) (1/mm^3^)	29.67 ± 6.87	26.17 ± 3.78
Trabecular number (Tb N) (1/mm)	3.198 ± 0.17	3.101 ± 0.40
Trabecular thickness (Tb Th) (mm)*	**0.148 ± 0.01**	**0.168 ± 0.01**
Trabecular spacing (Tb Sp) (mm)	0.272 ± 0.03	0.285 ± 0.04
**Bone material density (mg HA/cm**^**3**^)*	**845.4 ± 5.61**	**861.4 ± 9.59**
Structure model index (SMI)	−1.710 ± 0.13	−2.155 ± 1.02

There were no statistically significant differences in the other measured parameters (bone volume fraction (BV/TV), trabecular number (Tb.N), trabecular separation (Tb.Sp), connectivity density (CD), and structure model index (SMI)).

### Histomorphometry

**Fig. 3:**
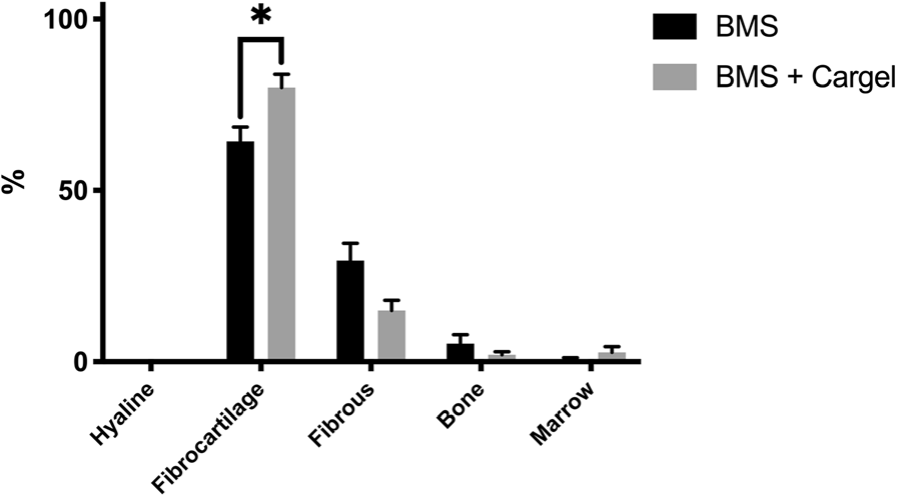
Mean fraction of hyaline tissue, fibrocartilage, and fibrous tissue (+ standard error of mean (SEM)) in the cartilage repair tissue. *n* = 11 for each group. BMS = bone marrow stimulation. Grey bar = CARGEL Bioscaffold + BMS, black bar = BMS only.* = *p* < 0.05

Histomorphometric analysis revealed significantly more fibrocartilage (80% vs 64%, *p* = 0.039) and a trend towards less fibrous tissue in the defects treated with CB (15% vs 30%, *p* = 0.052) (Fig. 3). Hyaline cartilage was only seen in one defect treated with CB and none treated with BMS alone. No significant differences were seen for bone (2% vs 5%, *p* = n.s. (not significant)), or marrow (3% vs 1%, *p* = n.s.). No significant differences were seen between proximal, lateral and distal, medial defects. On single slides centrally in the defects there were no significant differences in metachromasia on safranin-O staining between the two treatment groups (47% vs 37%, *p* = n.s.).

### Semiquantitative scoring

**Fig. 4:**
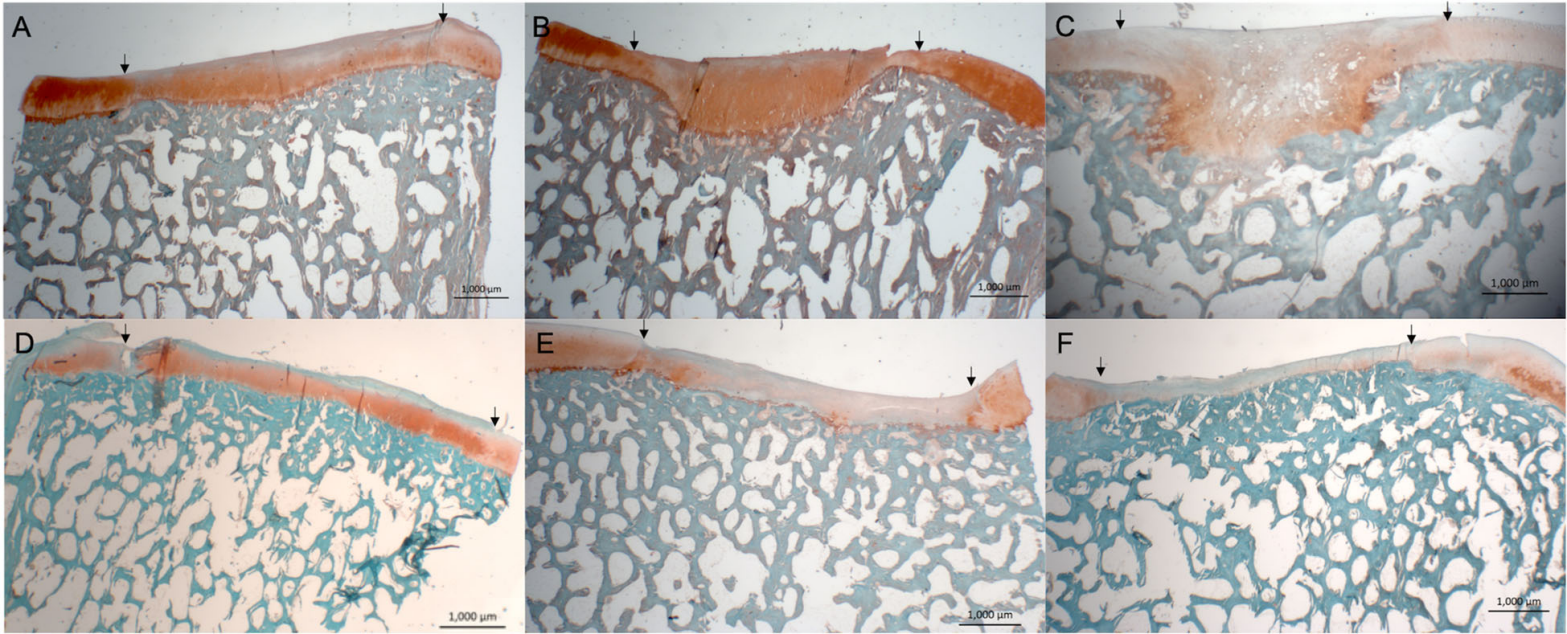
Safranin O staining; scale bars: 1000 μm. Magnification 12.5-fold. Defects treated with CB (A-C) and BMS (D-F). Images represent best (left), average (middle) and worst (right) repair. A larger extent of subchondral remodeling is seen in the CB treated defects, whereas there also seems to be a higher degree of metachromasia in CB treated defects

**Fig. 5:**
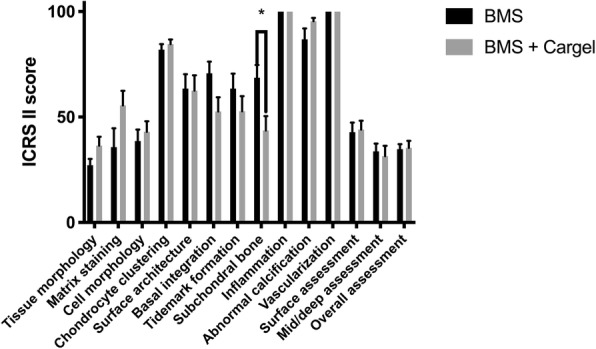
Mean International Cartilage Repair Society (ICRS) II scores (+ standard error of mean (SEM)). *n* = 11 for each group. Higher scores indicate better tissue. BMS = bone marrow stimulation. Grey bar = CARGEL Bioscaffold + BMS, black bar = BMS only. * = p < 0.05

**Fig. 6:**
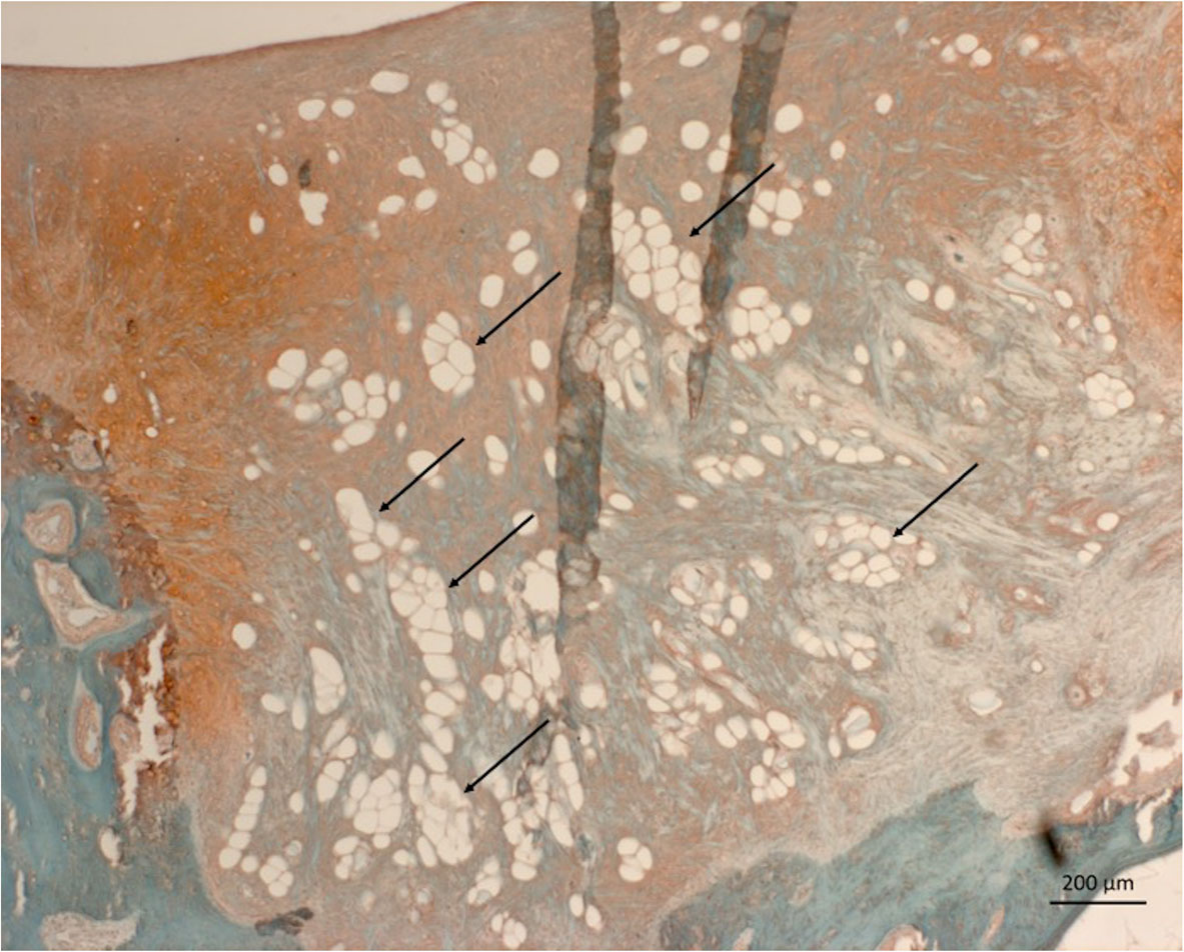
Safranin O staining; scale bar 200 μm. Magnification 50-fold. Infiltration of marrow (arrows), seen as fat, into a CB treated defect

Complete or almost complete filling was found histologically in all defects. Best, average and worst examples of both treatments are shown in Fig. 4. In general, ICRS II scores were good for chondrocyte clustering and abnormal calcification/ossification even though two defects in the BMS group had small osteophytes in the repair tissue area (Fig. 5). No significant differences were seen between proximal, lateral and distal, medial defects for any categories. Cartilage and subchondral bone histology were altered for both groups as observed on tissue morphology, matrix staining, cell morphology, surface architecture and tidemark formation. There were no differences between the overall assessments. “Subchondral bone abnormalities/marrow fibrosis” (44 vs. 69, *p* = 0.043) scored significantly lower in defects treated with CB. All defects had altered subchondral bone seen as small osteophytes or an irregular subchondral bone plate. Alterations in the subchondral bone plate ranged from almost complete restoration to intense remodeling with fibrovascular infiltration in the marrow space. Six of 11 defects treated with CB had pronounced changes in the subchondral bone, while this was only seen in one defect treated with BMS alone. Three defects in the CB group, in three different minipigs, had infiltration of fat into the defect area (Fig. 6). There was a trend towards a lower score on “Basal integration” in defects treated with CB, however, the difference was not statistically significant (53 vs 71, *p* = 0.069). Basal integration ranged from almost full basal integration to very little basal integration. There were no significant differences in any other subscales of the ICRS II score (Fig. 5). Inflammation and vascularization were not seen in any defect. There was a slight trend towards more matrix staining (56 vs 36, *p* = 0.184) and better tissue morphology (36 vs 27, *p* = 0.151) in the CB group.

### Immunohistochemistry

**Fig. 7:**
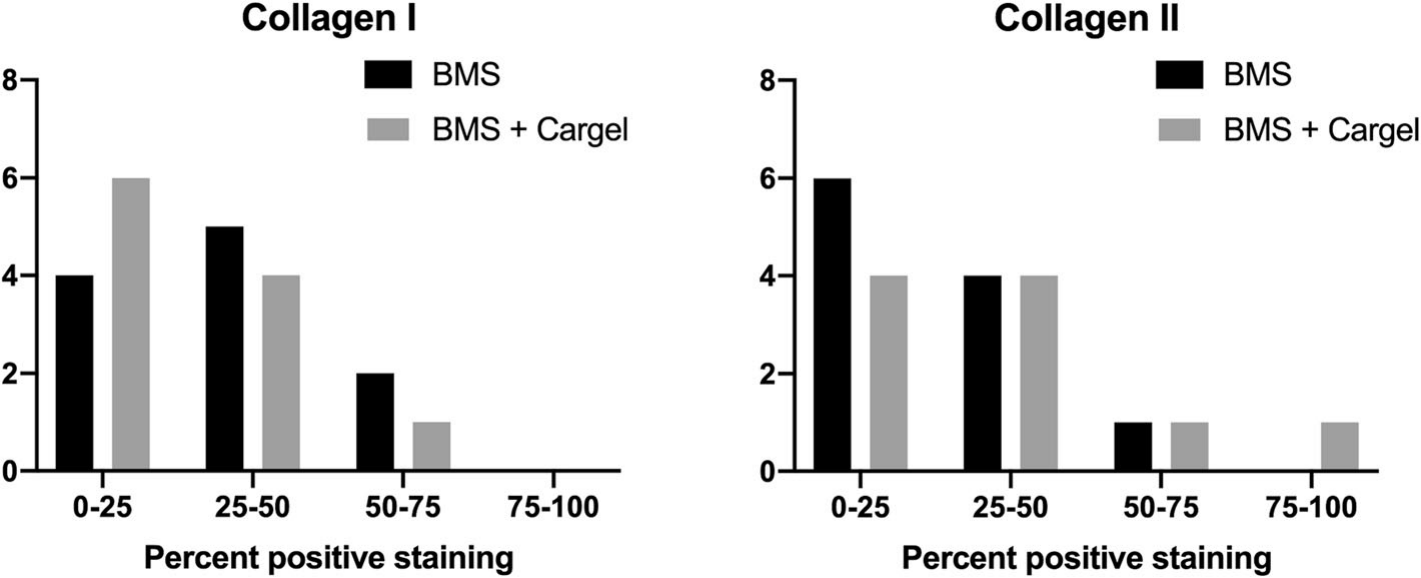
Graph showing quartiles of positive collagen I (left) and collagen II (right) staining. Grey bar = CARGEL Bioscaffold + BMS, black bar = BMS only. The y-axis represents the number of samples with the quartile of positive staining. In normal hyaline cartilage low amounts of collagen type I and high amounts of collagen type II is seen. BMS = bone marrow stimulation

**Fig. 8:**
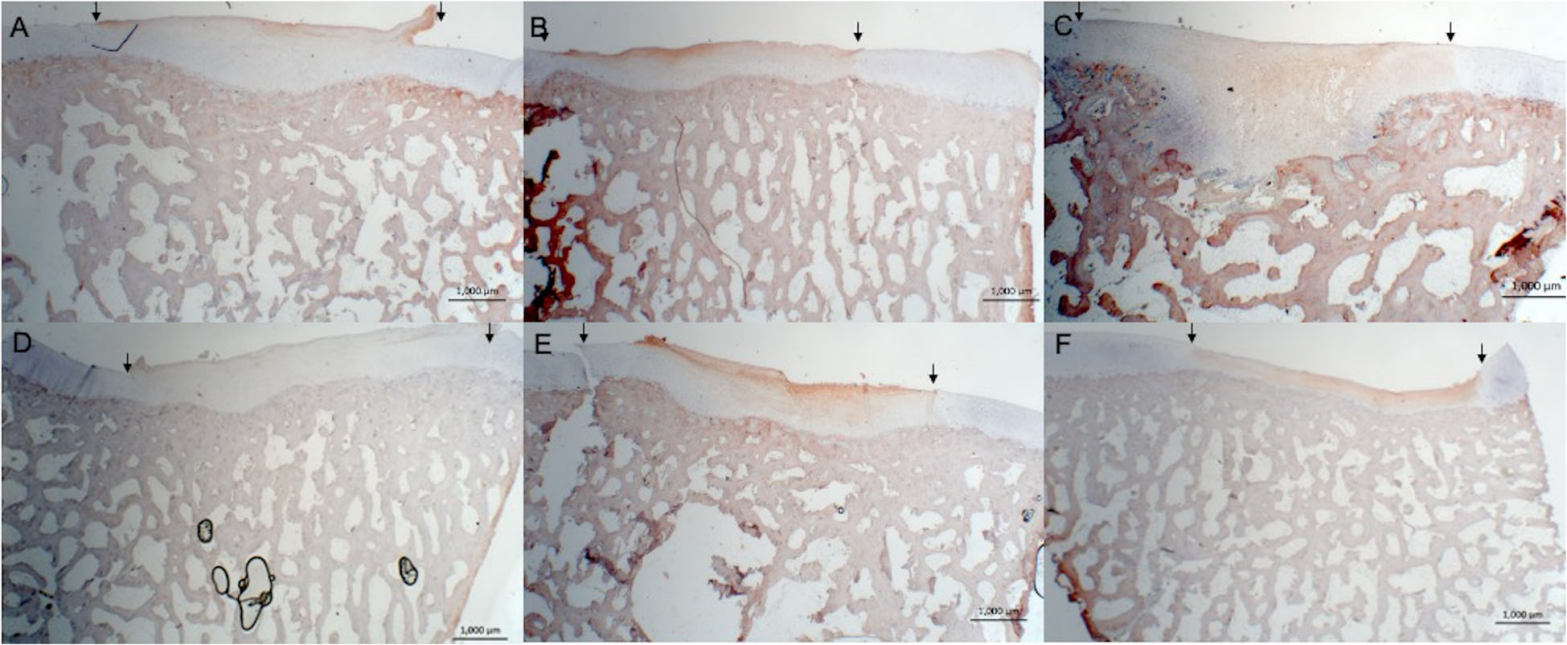
Collagen I staining; scale bar 1000 μm. Magnification 12.5-fold. Defects treated with CB (**a**-**c**) and BMS (**d**-**f**). Images represent 0–25% (left), 25–50% (middle) and 50–75% (right) positive collagen I staining

**Fig. 9:**
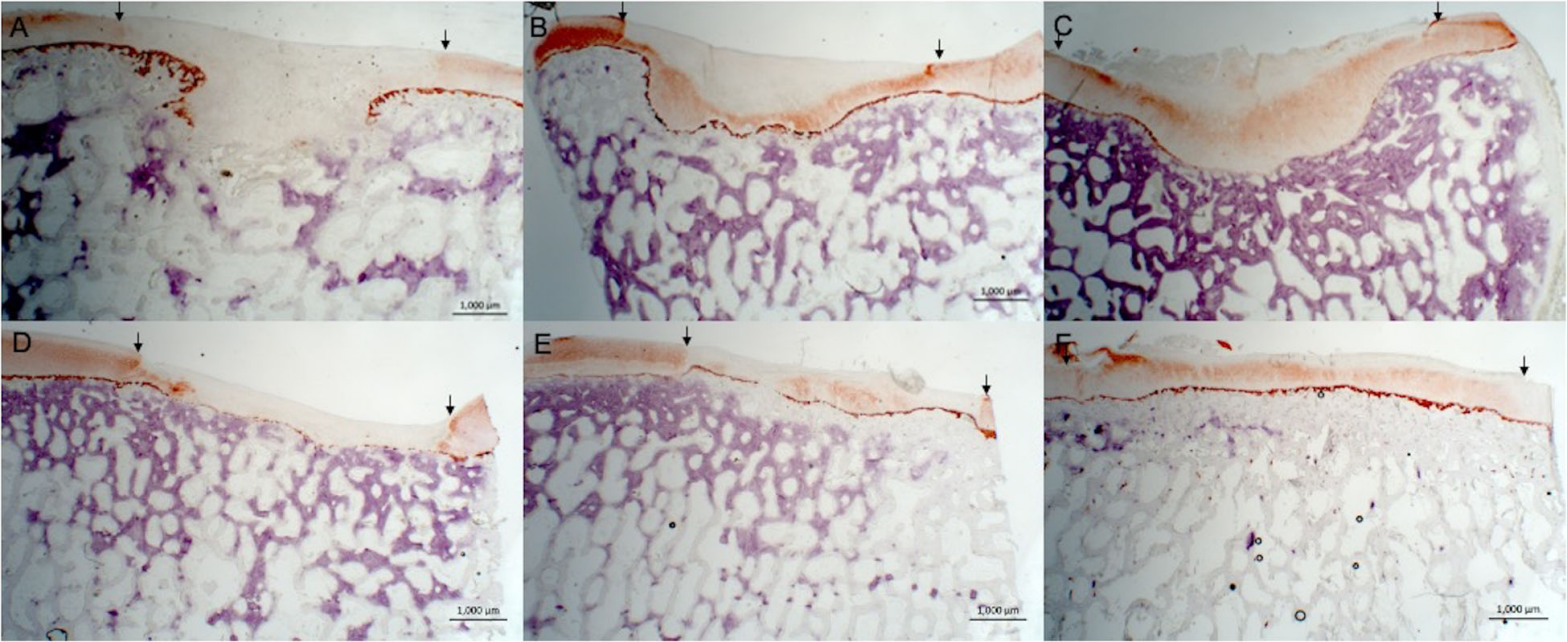
Collagen II staining; scale bar 1000 μm. Magnification 12.5-fold. Defects treated with CB (**a-c**) and BMS (**d**-**f**). Images represent 0–25% (left), 25–50% (middle) and 50–75% (right) positive collagen II staining

Most defects in both groups had < 50% positive staining for collagen type I or II (Fig. 7, 8, and 9). There was a trend towards less staining for collagen type I and more staining for collagen type II in defects treated with CB, however this was not statistically significant (*p* = 0.11 and *p* = 0.08, respectively).

## Discussion

The main findings of this study are that addition of CARGEL Bioscaffold to bone marrow stimulation led to a significant increase in fibrocartilage and a trend towards reduced fibrous tissue in the cartilage repair tissue. In addition, the subchondral bone had thicker trabeculae and higher mineralized bone tissue in defects treated with CB and BMS. CB seemed to improve repair tissue and induce a more pronounced subchondral remodeling. However, hyaline cartilage was not produced. Most ICRS II parameters and immunohistochemical stainings did not differ between the two treatment groups.

Only one other large animal study has been conducted with this chitosan hydrogel [8]. In that study a higher defect filling and a higher percentage of hyaline repair tissue in the CB group compared with a microfracture only group was found using an ovine model. In that study, however, it was assumed that tissue staining pink or red with safranin O was hyaline cartilage. Hoemann et al. reported 86% hyaline cartilage in the CB group and 71% in the microfracture group, which is in stark contrast to < 1% hyaline cartilage found in the repair tissue of both groups in the present study. We included single section histologic analysis using safranin-O to allow for comparison with the study by Hoemann et al. and found proportions of hyaline cartilage closer to what was reported in that study (47% and 37%) [8]. In the present study, however, we found no statistically significant differences between the groups. The limited fraction of hyaline cartilage in the present study is in line with other animal studies on cartilage repair [26–28]. In the present study we used polarized light and H&E staining to quantitatively determine whether the repair tissue was hyaline cartilage, fibrocartilage or fibrous tissue. Use of polarized light was not described by Hoemann et al. and this may have led to an insufficient distinction between fibrocartilage and hyaline cartilage [8, 24].

It is also noteworthy that Hoemann et al. observed the best repair on the femoral condyles, whereas the trochlear defects, as used in our study, had significantly less hyaline repair tissue [8]. Another study conducted on New Zealand White Rabbits also reported more hyaline tissue after 6.5 months in defects filled with CB compared to BMS and thrombin. Here tissue staining red or pink was also assumed to be hyaline cartilage. Defects treated with CB also scored higher on the O’Driscoll score than the control group [17]. No differences were seen between proximal/lateral and distal/medial defects. Shear forces on the repair tissue may theoretically differ at these sites and a study on BioCartilage showed differing outcome on distal and proximal defects on the lateral trochlear ridge in an equine model [29]. Therefore, site (medial or lateral) was accounted as a fixed effect in the mixed model analysis. However, because we found no differences between proximal/lateral and distal/medial defects, the results from all defects were counted together.

Clinical studies on CB have also showed an improvement in repair tissue quantity and quality on MRI and histological evaluation of biopsies of repair tissue, although a significant improvement in patient outcome after 5 years has not been seen [9, 10, 30].

A varying degree of remodeling was seen in subchondral bone beneath the defects. Subchondral remodeling was pronounced in 6 of 11 defects treated with CB, whereas it was mild in all other defects, except for one, treated with BMS. Remodeling of the subchondral bone is a known possible “side effect” to cartilage repair techniques such as BMS as well as autologous chondrocyte implantation (ACI) and involves both bone overgrowth and development of intralesional cysts [31, 32]. This can lead to subchondral edema, lack of graft integration and treatment failure. Remodeling of the subchondral bone is often seen in animal models of cartilage repair and is most likely due to a combination of osteoclast activation, altered mechanical loading and access of the synovial fluid to the subchondral bone [33–36]. A larger degree of subchondral remodeling in the CB treated defects may be due to a larger bioactive response of the subchondral bone due to the chitosan in the CB scaffold. A study on CB reported that the addition of CB to BMS delayed maturation and ossification of chondrogenic foci in drill holes [7]. The authors argued that this may be due to the increased recruitment of neutrophils and altered macrophage activation in drill holes beneath chitosan implants [6, 37]. Inflammation has been reported to delay callus formation in fracture repair models [38]^,^ but delayed healing may promote regeneration by progenitor cells and thereby hinder development of fibrous scar tissue, thus leading to a better and more mature repair tissue [39]. Similarly, a study by Chen et al. compared different types of BMS in a rabbit model and found that increased subchondral bone activation and remodeling improved cartilage repair [33]. This is also in line with the findings in the present study where we found an increased subchondral bone activity and a better repair tissue with CB treatment compared with BMS alone. It is possible that with longer follow-up the subchondral bone and cartilage repair tissue would have improved further.

The μCT analysis revealed thicker trabeculae in the CB group. The above-mentioned study by Marchand et al. of CB in New Zealand White Rabbits reported thicker trabeculae in defects with more residual holes after BMS [17]. Contrary to the findings in the present study, trabecular thickness was higher in the control group compared with CB, but the control group also had more residual drill holes after drilling. It can be speculated whether the thicker trabeculae is caused by a direct response elicited by the CB or whether it is an indirect response to the altered mechanical loading due to the pronounced alteration of the subchondral bone seen in more than half of the CB treated defects in our study [40]. The differences in the subchondral bone and subsequently the thicker trabeculae found in our study may be a result of faster bone turnover rates in New Zealand White Rabbits compared with Göttingen Minipigs [41].

The higher bone material density, however, points towards more mature bone in the spongiosa beneath the subchondral bone plate in the defects treated with CB. CB is a liquid before stabilization of the clot and has access to the bone and bone marrow through the drill holes. Residual chitosan may adhere to the calcified cartilage and bone and influence bone repair through increased cell recruitment [6, 8, 16].

Adipose tissue was present in three defects treated with CB. Adipose tissue has not been mentioned in any of the previous literature on CB [7, 8, 14, 15, 17, 30, 33, 34, 37, 42–45]. The presence of adipose tissue in the repair tissue area is of course an unwanted response to BMS. It has occasionally been seen in animal models of BMS [15, 37]. So, whether the presence of adipose tissue was related to CB is impossible to conclude. MSCs from the bone marrow are defined by their ability to undergo differentiation into different cell types such as chondrocytes, adipocytes, or osteocytes depending on humeral environment and this may explain the fat infiltration [46]. Chitosan has been shown to influence fat metabolism when used as dietary supplement and this effect could perhaps also be exerted by local application as in the present study [47].

A trend towards more collagen type II (indicative of hyaline cartilage) and less collagen type I (indicative of fibrous tissue/bone) was seen in CB treated defects. However, no significant differences were seen in the immunohistochemical stainings for collagen type I and II. The trend was, however, in line with the histomorphometric results showing more fibrocartilage in the CB treated defects. Staining for collagen type II generally correlated well with the metachromasia seen on Safranin O stained sections. While immunohistochemical staining for collagen type I and II can be an important tool in the analysis of cartilage repair tissue, limitations exist with regards to immunohistochemistry as a loss of antigenicity may occur and weak staining may be seen [48, 49]. This limited our ability to quantify the amount of positive staining accurately and was the reason for using quartiles. This, naturally, reduced the chances of finding significant differences. That most defects stained < 50% positive for collagen type II was in line with other studies with BMS in minipigs [20, 36, 50]. Some of the repair tissue did not stain positively for either collagen type I or type II and this may indicate the presence of an immature repair tissue containing other combinations of collagens [51, 52].

A strength of the study was that it was performed in a validated large animal model with comprehensive investigation of repair tissue quality and subchondral bone response including μCT, histomorphometry, histology, and immunohistochemistry. The existing literature on CB is primarily based on rabbit models, whereas literature on large animal models is sparse. With larger animal models, as the porcine, joint size and gait characteristics more closely resembles the human, but costs are significantly increased [18, 53, 54].. This offers great translational value, but also adds limitations due to the costs. Nevertheless, using a validated, large animal model these comprehensive repair tissue analyses can deliver data that are not possible to obtain in clinical studies. The treatment tested has already been used in the clinic for a few years and has proven safe and shown promising results with regards to patient outcome, repair tissue quality, and quantity [9, 12, 30]. The present study therefore adds to the understanding of the promising clinical outcomes with findings of increased fibrocartilage tissue formation and more repair tissue-supportive subchondral bone remodeling. It must though again be emphasized that no true hyaline cartilage was observed in repair tissues. Furthermore, large variations were seen in outcome on several parameters. This is often seen in large animal studies and may be an inherent limitation of large animal studies and is important to take into account when determining the number of animals/defects pr. treatment group in in vivo studies [21, 22, 28, 35, 50, 53]. Great variations in outcome with cartilage repair treatments is also seen in the clinical setting, but here it can, to some degree, be explained by patient (age, sex, BMI) and lesions (site, size) demographics.

Limitations to our study are that we only had one time point of evaluation making us unable to observe the temporal changes and degradation of the scaffold. The repair tissue may furthermore require more than 6 months for maturation [18]. The choice of only a single time point was mainly cost related. The follow-up period of 6 months is naturally significantly shorter than that used in clinical studies, but is considered sufficient for organized cartilage repair in minipigs [55, 56]. Other studies have, however, pointed towards changes in the biological repair between 6 and 12 months [18, 57]. Other limitations of the study include a lack of mechanical testing, which could have provided insight into the biomechanical properties of the repair tissue and the lack of immobilization after surgery as recommended in the clinic. Rehabilitation is highly important clinically with reduced loading of areas of repair recommended. However, immobilization is not possible in Göttingen minipigs [20, 58]. This may lead to an overload of repair tissue and a reduced healing response. An untreated control group was not included as the defects were of critical size, which are well-documented to fill with mainly fibrous tissue in Göttingen minipigs [18, 19, 56].

In conclusion, use of CARGEL Bioscaffold in combination with bone marrow stimulation did not lead to formation of hyaline cartilage but does seem to induce an improved repair tissue and a more pronounced subchondral bone remodeling compared with bone marrow stimulation alone, which may be a predictor for improved repair tissue.

## Supplementary information

**Additional file 1 Supplementary figure 1.** Macroscopic images of the 6 knees treated with bone marrow stimulation alone.

**Additional file 2 Supplementary figure 2.** Macroscopic images of the 6 knees treated with bone marrow stimulation in combination with CARGEL Bioscaffold.

## Data Availability

The datasets used and/or analysed during the current study are available from the corresponding author on reasonable request.

## Abbreviations

BMS: Bone marrow stimulation
Mfx: Microfracture
CB: CARGEL Bioscaffold
ICRS: International Cartilage Repair Society
ACI: Autologous chondrocyte implantation
