# Supplementary figures and images for: CARGEL Bioscaffold improves cartilage repair tissue after bone marrow stimulation in a minipig model
Source: J Exp Orthop. 2020 May 8;7:26. doi: 10.1186/s40634-020-00245-7 (PMC7210369; doi:10.1186/s40634-020-00245-7)

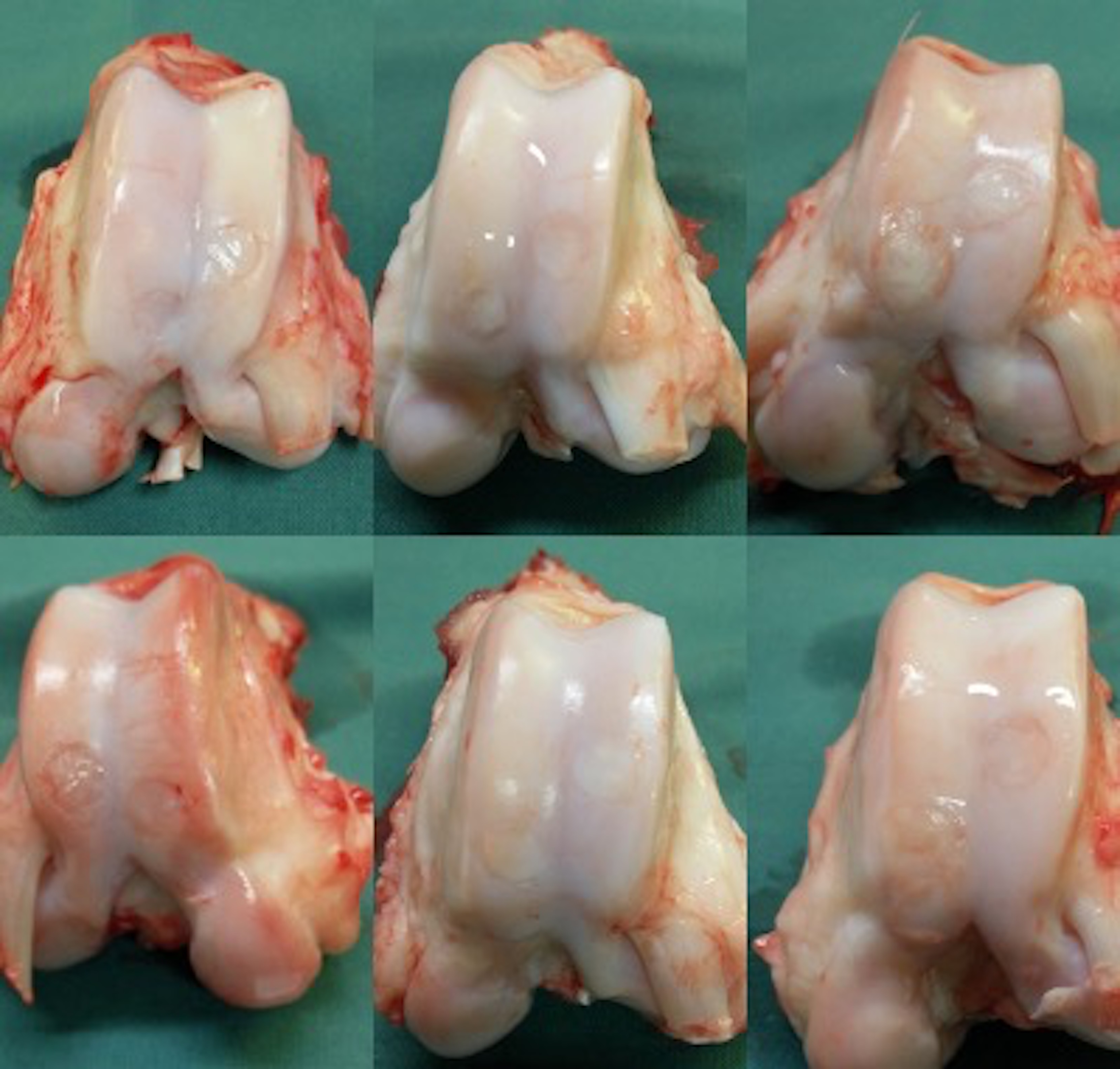

Supplement: Supplementary file 1 — Additional file 1 Supplementary figure 1. Macroscopic images of the 6 knees treated with bone marrow stimulation alone. [file 40634_2020_245_MOESM1_ESM.jpg]

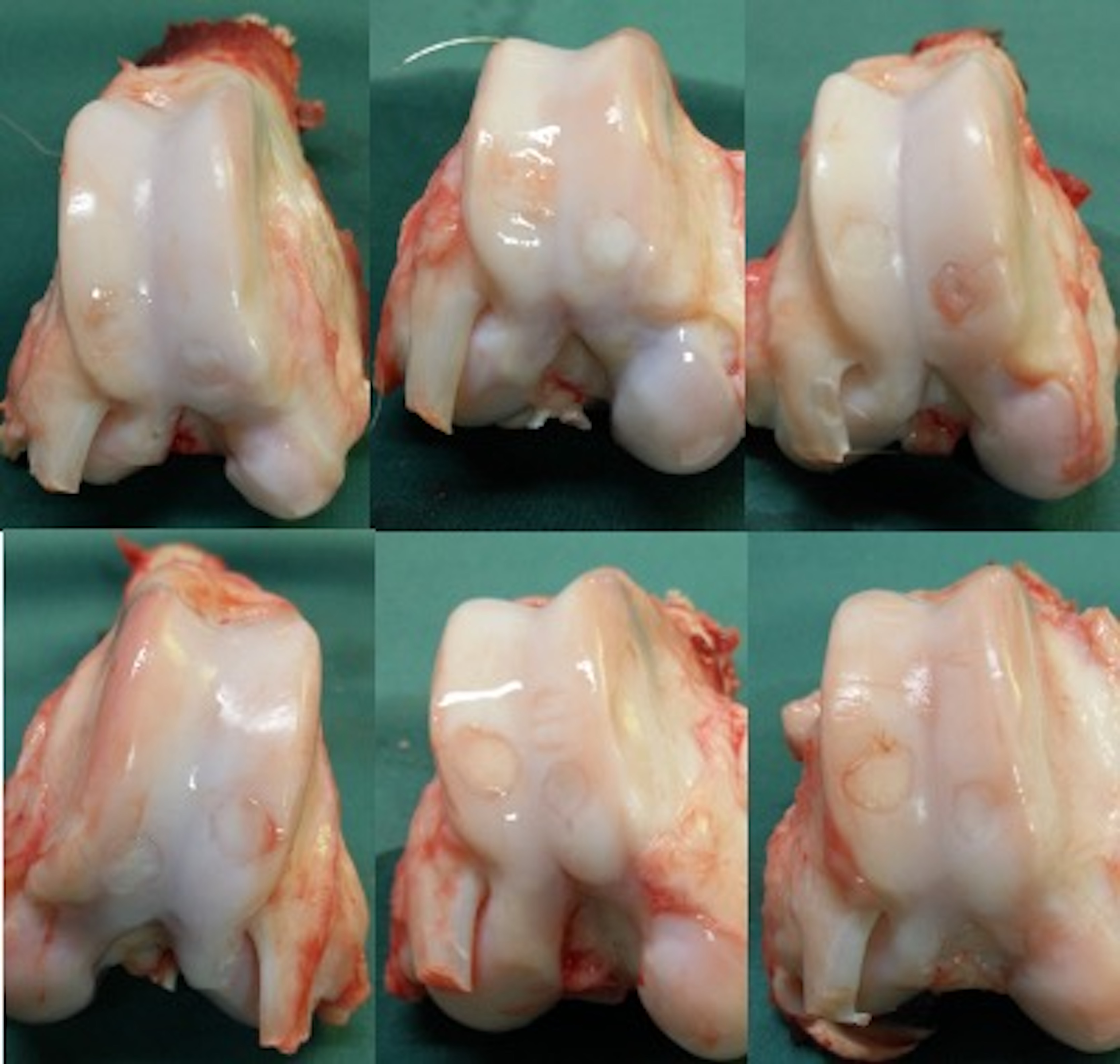

Supplement: Supplementary file 2 — Additional file 2 Supplementary figure 2. Macroscopic images of the 6 knees treated with bone marrow stimulation in combination with CARGEL Bioscaffold. [file 40634_2020_245_MOESM2_ESM.jpg]
